# Proanthocyanidins Inhibit the Transmission of Spinal Pain Information Through a Presynaptic Mechanism in a Mouse Inflammatory Pain Model

**DOI:** 10.3389/fnins.2021.804722

**Published:** 2022-02-03

**Authors:** Hongwei Fan, Zhenyu Wu, DaYu Zhu, Junxiang Gu, Mang Xu, Mingzhe Zhang, Haokai Duan, Yunqing Li, Tao Chen

**Affiliations:** ^1^Department of Human Anatomy, Xuzhou Medical University, Xuzhou, China; ^2^Department of Anatomy, Histology and Embryology, K. K. Leung Brain Research Centre, Fourth Military Medical University, Xi’an, China; ^3^Epilepsy Center of Xijing Hospital, Fourth Military Medical University, Xi’an, China; ^4^Department of Neurosurgery, The Second Affiliated Hospital of Xi’an Jiaotong University, Xi’an, China; ^5^Department of Anatomy, Basic Medical College, Dali University, Dali, China

**Keywords:** proanthocyanidins, inflammatory pain, spinal cord, excitatory postsynaptic currents, mice

## Abstract

Inflammatory pain is one of the most common symptoms of clinical pain that seriously affects patient quality of life, but it currently has limited therapeutic options. Proanthocyanidins, a group of polyphenols enriched in plants and foods, have been reported to exert anti-inflammatory pain-alleviating effects. However, the mechanism by which proanthocyanidins relieve inflammatory pain in the central nervous system is unclear. In the present study, we observed that intrathecal injection of proanthocyanidins inhibited mechanical and thermal pain sensitivity in mice with inflammatory pain induced by Complete Freund’s Adjuvant (CFA) injection. Electrophysiological results further showed that proanthocyanidins inhibited the frequency of spontaneous excitatory postsynaptic currents without affecting the spontaneous inhibitory postsynaptic currents or the intrinsic properties of parabrachial nucleus-projecting neurons in the spinal cord. The effect of proanthocyanidins may be mediated by their inhibition of phosphorylated activation of the PI3K/Akt/mTOR pathway molecules in dorsal root ganglia neurons. In summary, intrathecal injection of procyanidin induces an obvious anti-inflammatory pain effect in mice by inhibiting peripheral excitatory inputs to spinal neurons that send nociceptive information to supraspinal areas.

## Introduction

Inflammatory pain is caused by chemical or physical stimulation of damaged tissue (surgery, osteoarthritis or trauma, etc.). In the condition of inflammatory pain, the released chemical mediators act on the receptors located in the peripheral process of dorsal root ganglia (DRG) neurons and increase the afferent firing from their central process to the spinal dorsal horn, leading to the hyperactivity of spinal nociceptive neurons and inducing allodynia and hyperalgesia (Demir et al., 2013; Muley et al., 2016). In the spinal cord, neurons that send nociceptive information to supraspinal areas are primarily located in lamina I of the dorsal horn, 95% of which project to the parabrachial nucleus (PBN). This type of projection neuron is also an important afferent target for the transmission of enhanced nociceptive information (Todd, 2010).

Proanthocyanidins are oligomers or polymers composed of units of flavanols extracted from cherry, grape seeds, cocoa, and other plants (Chacón et al., 2009). Their protective effects in nervous system diseases have received increasing attention in recent years. In addition to their widely known antioxidant, antiapoptotic, and antiallergic effects (Martinez-Micaelo et al., 2012, 2015), their roles in alleviating inflammatory pain have gradually been recognized. It has been reported that proanthocyanidins reduce the amount of abdominal writhing induced by intraperitoneal (*i.p.*) injection of acetic acid and decrease the duration of formalin-induced paw licking (Cordeiro et al., 2016), by peripherally inhibiting the inflammatory exudation (Sobrinho et al., 2017). Intra-articular injection of proanthocyanidins also inhibits the expression of inflammasomes in macrophages and relieves arthritis (Liu et al., 2017). However, these results primarily indicate that proanthocyanidins have anti-inflammatory effects by acting on peripheral tissues. Although Pan et al. (2018) reported that proanthocyanidins produce behavioral analgesia by inhibiting activated matrix metalloproteinase (MMP)-9 and MMP-2 in the spinal cord of mice with neuropathic pain, whether proanthocyanidins relieve inflammatory pain and the possible mechanism in regulating nociceptive information transmission in the central nervous system remain elusive.

Therefore, in the present study, we investigated whether and how proanthocyanidins induce antinociceptive effects at the spinal cord level in mice with inflammatory pain. We found that intrathecal administration of proanthocyanidins increased the mechanical and thermal pain threshold in mice 7 days after Complete Freund’s Adjuvant (CFA) injection. Bath application of proanthocyanidins reduced excitatory peripheral inputs to PBN-projecting spinal cord neurons. Western blotting analysis further revealed that proanthocyanidins may inhibit the phosphorylated activation of PI3K/Akt/mTOR pathway molecules in the DRG neurons. Our work shows for the first time that the anti-inflammatory pain effect and mechanism of proanthocyanidins occur in the spinal cord of mice.

## Materials and Methods

### Animals

The animals used in the experiment were all adult male C57BL/6 mice, 8–12 weeks of age, weighing 18–30 g. Forty mice were used for behavioral tests, 32 mice were used in Western blotting, and 65 mice were used in whole-cell patch experiments. All mice were raised in a pathogen-free environment with a constant temperature of 23°C, humidity of 50 ± 10%, 12 h light/dark cycle, and adequate food and water. The use agreement for experimental animals was approved by the Animal Care and Use Committee for Research and Education of the Fourth Military Medical University (Xi’an, China). All experiments were performed in a single-blind manner. The experimenters who collected the raw data were not aware of group allocation.

### Drugs

Proanthocyanidins (natural extracts, CAS number: 4852-22-6, Molecular formula: C30H26O13, Molecular weight: 594.52) were purchased from Shanghai Yuanye Biotechnology (Shanghai Yuanye Biological Technology Co. Ltd.). The solution was dissolved in artificial cerebrospinal fluid (ACSF) to produce the original solution at a concentration of 8.41 mM, that is, 5 mg/ml. The final concentration was diluted in ACSF for behavioral and electrophysiological experiments.

### Model of Complete Freund’s Adjuvant-Induced Inflammatory Pain

Before the animal model was established, the mice were fully adapted to the environment and were subjected to mechanical pain sensitivity tests 1 day in advance to monitor the baseline pain threshold. After the mice were anesthetized by 1.5% isoflurane in 100% O_2_ at 1.5 L/min, 10 μl of CFA (obtained from Sigma, F5881-10 × 10 ml) was injected into the left hind paw of the CFA group using a microinjector, and the needle was left in place for 10 s after injection to prevent CFA overflow. The saline group was injected with the same amount of normal saline, and the needle was kept in place for the same time. From 30 min to 1 h after injection, redness and swelling were observed in the hind feet of the CFA group, while no significant changes were observed in the hind feet of the saline group. After 1 day, mechanical pain sensitivity was tested in both groups. The mechanical pain sensitivity threshold of mice in the CFA group was significantly decreased compared to that before the injection of CFA, while the mechanical pain sensitivity threshold of mice in the saline group was not significantly changed compared to that before the injection of saline, indicating that the inflammatory pain model of CFA was successfully established.

### Intrathecal Injection

Mice were placed in open-hole 50-ml centrifuge tubes to limit head movement to the tube, and the tail and waist were exposed. According to Hylden and Wilcox’s method (Hylden and Wilcox, 1980), 20 μg of proanthocyanidins dissolved in ACSF was injected into the L5 and L6 regions of mice using an insulin microinjector after removing the hair from the waist and tail, and the saline group was injected with the same amount of ACSF as the control experiment. When the tip of the needle accurately entered the intervertebral space, rapid movement of the mouse tail could be observed to demonstrate the success of needle insertion into the sheath, and clear and colorless cerebrospinal fluid could be seen during withdrawal. The injection speed was slow and uniform, and the needle was kept in place for 10 s after the injection to prevent liquid efflux.

### Behavioral Testing

Behavioral testing was performed referring to Yin’s method (Yin et al., 2020). Before the mechanical pain sensitivity study, mice were placed in separate plexiglass boxes covered with clear plastic sheets. After the mice were adapted to the box for 30–60 min, we used a set of calibration intact Von Frey filaments (scale range is 0.04 and 0.07, 0.16 and 0.40, 0.60 and 1.00, and 1.40 and 2.00 g) to stimulate mice using the paw foot measure do determine their paw withdrawal mechanical threshold (PWMT). Von Frey filaments were applied vertically to the plantar surface of the hind paws of mice with sufficient force until the Von Frey filaments bent or the mice exhibited pain manifestations such as paw retraction and foot licking, which were recorded as positive responses. Each grade of Von Frey filament scale was stimulated five times in each hind paw with an interval of 5 min, and three positive responses were observed as the PWMT. If no positive response occurred after >3 times, the next larger Von Frey filament was applied. To prevent injury during the test, the maximum intensity of Von Frey filaments was 2.00 g.

To determine thermal sensitivity in mice, we followed Rashid’s method (Rashid et al., 2006). The paw withdrawal thermal latency (PWTL) of the mice was measured. Each mouse was placed in a separate plexiglass box and placed on a thermostatically elevated glass plate upon a thermal radiation stimulator. After the mice were adapted for 30 min–1 h, each hind paw was exposed to constant heat radiation five times at an interval of 10 min, and the average value of the latent time of paw retraction was taken as the heat pain sensitivity value. To prevent injury to the rear paw, each stimulation time was <20 s.

An open field test was performed to determine whether intrathecal injection would affect the locomotor ability of the mice. In a 50 cm × 50 cm square box, the underside is white. Mice were placed in the laboratory in advance and allowed to adapt to the environment for 1 h, and 75% alcohol was used to eliminate the odor of the boxes and environment used in the experiment. Each mouse was placed separately into the center of each square box, and the video recording began for 15 min. Automatic analysis software was used to analyze the locomotor route of the mice.

We performed the rotating rod test in mice to test the motor coordination ability of mice after intrathecal injection of proanthocyanidins. Before the experiment, the mice were placed in the test chamber for 30 min to adapt and then placed on the rotating rod in the opposite direction of rotation. The rotary bar rotates at speeds from 5 to 30 r/min. The time until the mice fell from the bar was recorded. The test was repeated three times for each mouse with an interval of 10 min, and the average of the results was taken.

### Brain Stereotaxic Injection

To label the noxiously transmitting neurons in the superficial neurons of the dorsal horn of the spinal cord, we injected the retrograde tracer TMR into the PBN site of the mouse head. After intraperitoneally injecting Ketamine/Xylazine mixture in 100 and 10 mg/kg body weight, respectively, the mice were deeply anesthetized and fixed in the prone position on a stereotaxic apparatus (NARISHIGE Scientific Instrument Las, Tokyo, Japan). The microinjector (1 μl, Hamilton, NV, United States) was introduced into the PBN according to the following coordinates: 5.1 mm after the anterior fontanelle, 1.25 mm outside the midline, and 3.4 mm below the surface of the skull (Bai et al., 2020). A small hole was drilled into the skull above the PBN position, the broken bone was removed, and a hemostatic sponge was used to remove the oozing blood. The trace injector [0.15 μl 10% tetramethylrhodamine (TMR), D3308, 3000 MW, Molecular Probe, Eugene, OR, United States] was injected into the PBN coordinate position. After injection for 15 min, the needle was kept in the original position for another 30 min. After surgery, the mice were placed on a heating pad to keep their body temperature at approximately 37°C until they woke up at which point they were treated with antibiotics in the abdominal cavity and at the site of the head wound. The electrophysiological experiment was started on the seventh day after the surgery was completed.

### Immunofluorescent Histochemical Staining

To enable the observation of TMR-labeled PBN projected neurons recorded by whole-cell patch clamp, 1% biocytin was added to the intracellular solution. After recording, the spinal cord sections were fixed in precooled 4% paraformaldehyde for 4–6 h and then washed with 0.01 mol/L PBS solution containing 0.5% Triton X-100 (pH 7.4) three times for 10 min each. In addition, avidin conjugated with Alexa-488 fluorophore (obtained from Invitrogen, S11223) was added to PBS at a dilution ratio of 1:1000 to incubate the sections for 4–6 h. Finally, the slices were washed with PBS three times for 10 min each, fixed on clean slides, and sealed with fluorescent tablets. The sections were then observed under a confocal laser scanning microscope (FV-1000, Olympus, Tokyo, Japan), and an appropriate laser beam [Alexa 488 (excitation at 488 nm; emission 510–530 nm), Alexa 594 (excitation 543 nm; emission 590–615 nm), Fluoview software (Olympus)] was used to capture digital images.

### Western Blotting

Under isoflurane anesthesia (1.5% isoflurane in 100% O_2_ at 1.5 L/min), mice in the saline, CFA, and proanthocyanidin treatment groups were decapitated and sacrificed in extraction buffer containing 100 mM Tris, pH 7.4, 10 mM EDTA, and 2 mM PMSF. The samples were derived from eight mice and then homogenized and centrifuged. Samples containing 75 μg protein were loaded onto 12% acrylamide gels using a Bio-Rad Mini system. The transfer device was used to electrotransfer gels onto nitrocellulose membranes at 120 V for 1.5 h. Membranes were treated with a blocking solution containing 5% skimmed milk powder at room temperature for 2 h and washed followed by the addition of primary antibody: rabbit anti-PI3K (#4292, RRID:AB_329869), p-PI3K [#17366 (Sun et al., 2020)], PKA (#5842, RRID:AB_10706172), PKC (#9368, RRID:AB_10693777), p-PKC (#9378, RRID:AB_2168217), Akt (#9272, RRID:AB_329827), p-Akt (#9271, RRID:AB_329825), NF-κB (#3035, RRID:AB_330564), p-mTOR (#5536, RRID:AB_10691552), and mouse anti-mTOR (#2927, RRID:AB_2259936) (obtained from Cell Signaling Technology, diluted 1:1000 in PB containing 0.3% Triton X-100, Xi’an Kehao Biological Engineering Co. Ltd.) or mouse-β-actin (#A1978) (obtained from Sigma, diluted 1:5000) overnight at 4°C. Membranes were then washed and incubated with horseradish peroxidase-conjugated, anti-mouse (#ZB-2305) and anti-rabbit (#ZB-2301) secondary antibody diluted 1:5000 (Beijing Zhongshan Biological Technology Co. Ltd.) at RT for 2 h. Antibodies were detected using a chemiluminescence reagent kit (Xi’an Zhongtuan Biotechnology Co. Ltd.). The optical density of the bands was quantified using Bio-Rad Image Lab 5.1 (ChemiDocMXRS, Bio-Rad, United States). In control groups, all experimental procedures for immunostaining were similar but replaced the primary antibodies with normal serum. We found no immunostaining positive results in those experiments.

### Electrophysiological Studies

#### Spinal Cord Slice Preparation

We used whole-cell patch clamp to record spontaneous EPSCs and IPSCs of labeled PBN projective nociceptive neurons in the superficial dorsal horn in mice with lumbar enlargement. Before the whole-cell patch-clamp experimental study, we prepared mouse spinal cord sections according to Yu’s method (Yu et al., 2019). The mice were anesthetized by isoflurane anesthesia (1.5% isoflurane in 100% O_2_ at 1.5 L/min). After decapitation, the lumbar spine of the mice was rapidly cut and placed in precooled ASCF containing 95% O2 + 5% CO2. The ACSF for dissection contained the following (in millimolar): 252 sucrose, 2.5 KCl, 6 MgSO4⋅7H2O, 1.2 NaH2PO4, 26 NaHCO3, 0.5 CaCl2, and 10 D-glucose. Thoracolumbar laminectomy was performed using delicate surgical instruments to remove the L4-5 spinal cord and remove the dura, pia, and arachnoid meninges. A spinal cord vibration microtome (Leica VT1200S, Heidelberg, Nussloch, Germany) tray was used with an amplitude of 0.65 mm/s, speed of 0.20 mm/s, coronary section of the spinal cord, and a slice thickness of 300–400 μm. Then, the sections were incubated in oxygen-containing ACSF at room temperature for at least 1 h (in mmol/L: 124 NaCl, 2 CaCl2⋅H2O, 2.5 KCl, 1 MgSO4, 1 NaH2PO4, 37 D-glucose). The osmotic pressure was 310 mOsm.

#### Whole-Cell Patch-Clamp Recordings

Spinal cord sections were continuously infused with oxygen-containing ACSF at room temperature at a rate of 2.5–5 ml/min in a recording chamber, and the experimental operation was performed. Microelectrodes (6–9 Ω) were injected with intracellular fluid for experimental records. The injected intracellular fluid was divided into two types (in mmol/L): (1) a potassium gluconate-based solution containing 120 K^+^-glucose, 5 NaCl, 1 MgCl_2_, 10 HEPES, 0.2 EGTA, 2 MgATP, 0.1 Na_3_GTP, 10 phosphocreatine (Adjust pH using KOH to 7.2, 290 mOsm) and (2) a cesium mesylate-based solution containing 122 CSMeSO_3_, 3.7 NaCl, 20 HEPES, 10 BAPTA, 0.2 EGTA, 0.3 MgATP, 0.3 Na_3_ATP, 5 TEA-Cl, 5 QX314-Br (CsOH adjusted pH to 7.2, 290 mOsm). PBN-projecting neurons with TMR labeling were recorded in laminar I of the dorsal horn of the spinal cord. These neurons were visualized using a microscope equipped with infrared differential interference contrast optics. The neurons were voltage-clamped at −60 mV using whole-cell adsorption mode. Spontaneous excitatory postsynaptic currents (sEPSCs) were recorded by holding the cell membrane potential at −60 mV, and spontaneous inhibitory postsynaptic currents (sIPSCs) were recorded when holding at 0 mV. In two consecutive stimuli (interval 50 ms) pair pulse ratio (PPR) experiments, a bipolar stimulation electrode connection isolation current stimulator was used [Natus Medical Incorporated (nasdaq: PCLN – news, L6H5S1, Canada)] at an intensity of 20 μA. The ratio of the EPSC amplitude generated by the second stimulus to the EPSC amplitude generated by the first stimulus was calculated as the PPR value.

The above experiments guaranteed a sealing resistance >2 GΩ and a series resistance <35 MΩ; if series resistance changes during recording by >15%, the record result was discarded. We used a Multiclamp 700B amplifier (Axon Instruments, Foster City, CA, United States) to record all signals. Records and analyses of data used pCLAMP 10.2 (Axon Instruments) and mini-Analysis Program (Synatosoft Inc., NJ, United States). To enable observation of TMR-labeled PBN-projecting neurons, 0.5% biocytin was added to the intracellular solution.

### Statistical Analysis

**FIGURE 1:**
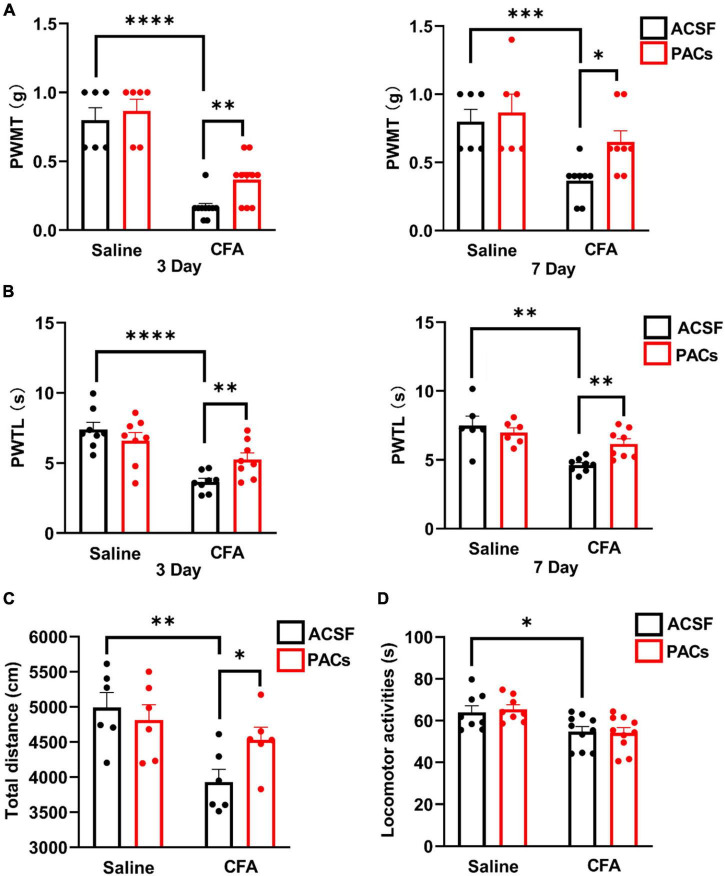
Intrathecal injection of proanthocyanidins increased mechanical and thermal pain thresholds in mice with CFA injection. Intrathecal injection of proanthocyanidins on the third and seventh day post-surgery increased the paw withdrawal mechanical threshold (PWMT) **(A)** and paw withdrawal thermal latency (PWTL) **(B)** of mice in the CFA group. **(C)** Intrathecal injection of proanthocyanidins increased the movement distance of mice in the CFA group. **(D)** Intrathecal injection of proanthocyanidins had no effect on the motor function of mice in either the CFA or saline group in the rotary rod test. *P < 0.05; ^**^P < 0.01; ^***^P < 0.001; ^****^P < 0.0001. PWMT, paw withdrawal mechanical threshold; PWTL, paw withdrawal thermal latency; CFA, Complete Freund’s Adjuvant; ACSF, artificial cerebrospinal fluid; PACs, proanthocyanidins.

**FIGURE 2:**
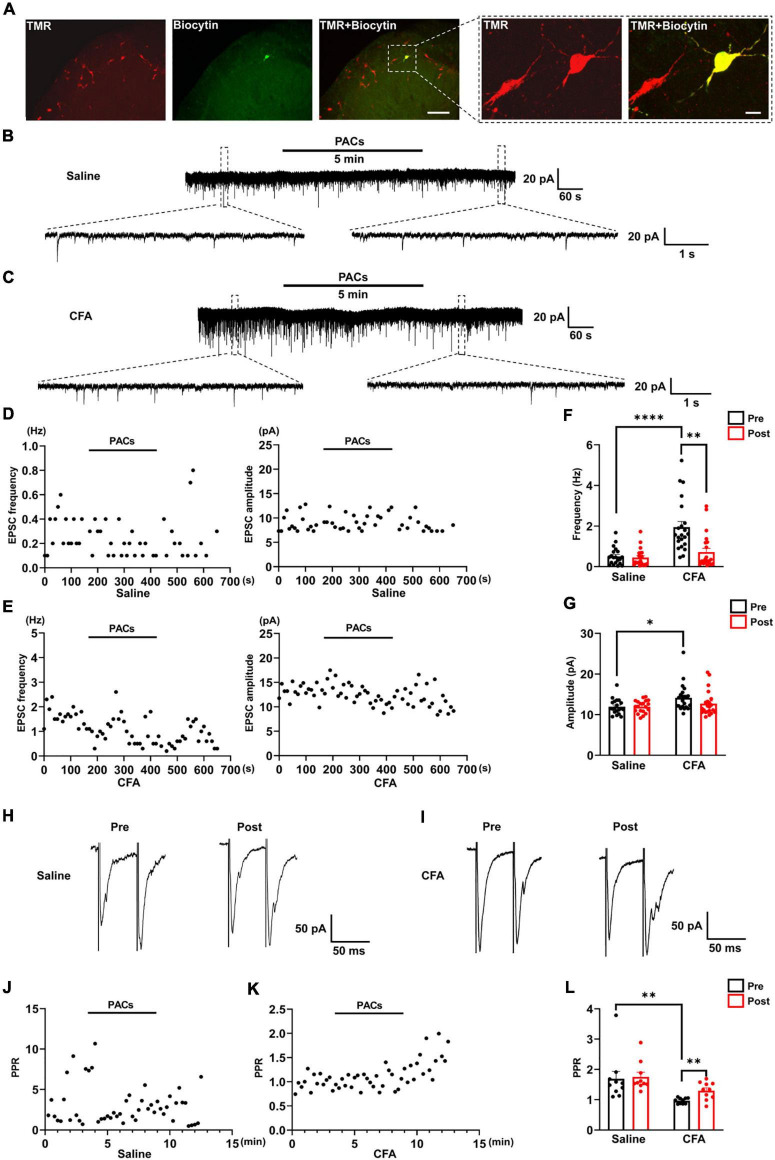
The effect of proanthocyanidins on sEPSCs of PBN-projecting neurons in the spinal dorsal horn. **(A)** Double-labeling immunohistochemistry staining confirmed that all recorded neurons were TMR-positive retrogradely labeled neurons (red) those distributed in laminar I of the left spinal cord. The rectangle area was enlarged in the right panel. The scale bars equal to 150 μm in the left panel images and 30 μm in the right panel images. Representative electrophysiological samples **(B)** and scatter plots **(D)** showing sEPSCs with bath application of proanthocyanidins (50 μM) in the saline group. Representative electrophysiological samples **(C)** and scatter plots **(E)** showing sEPSCs with bath application of proanthocyanidins in the CFA group. **(F)** Application of proanthocyanidins decreased the frequency of sEPSCs in the CFA but not saline group. **(G)** Application of proanthocyanidins had no effect on the amplitude of sEPSCs in the CFA or saline group. Representative electrophysiological samples **(H)** and scatter plots **(J)** showing the PPR with bath application of proanthocyanidins in the saline group. Representative electrophysiological samples **(I)** and scatter plots **(K)** showing the PPR with bath application of proanthocyanidins in the CFA group. **(L)** Application of proanthocyanidins increased the PPR in the CFA but not saline group. *P < 0.05; ^**^P < 0.01; ^****^P < 0.0001. TMR, tetramethylrhodamine; CFA, Complete Freund’s Adjuvant; PACs, proanthocyanidins; PPR, paired-pulse ratio.

**FIGURE 3:**
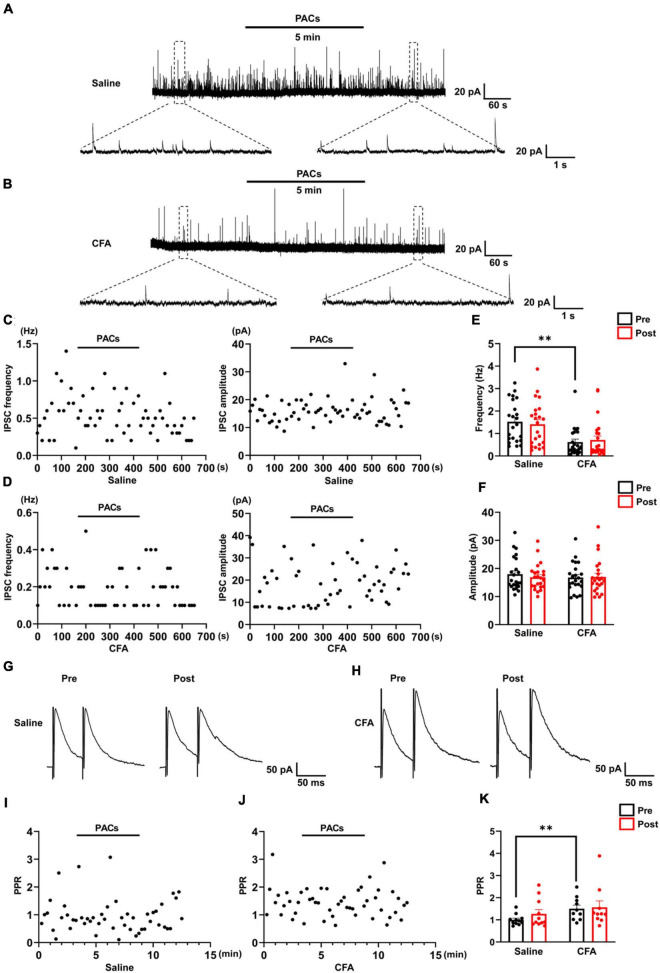
The effect of proanthocyanidins on sIPSCs of PBN-projecting neurons in the spinal dorsal horn. Representative electrophysiological samples **(A)** and scatter plots **(C)** showing sIPSCs with bath application of proanthocyanidins (50 μM) in the saline group. Representative electrophysiological samples **(B)** and scatter plots **(D)** showing sIPSCs with bath application of proanthocyanidins in the CFA group. Application of proanthocyanidins had no effect on the frequency **(E)** and amplitude **(F)** of sIPSCs in the CFA or saline group. Representative electrophysiological samples **(G)** and scatter plots **(I)** showing the PPR with bath application of proanthocyanidins in the saline group. Representative electrophysiological samples **(H)** and scatter plots **(J)** showing the PPR with bath application of proanthocyanidins in the CFA group. **(K)** Application of proanthocyanidins had no effect on the PPR in the CFA or saline group. ^**^P < 0.01. CFA, Complete Freund’s Adjuvant; PACs, proanthocyanidins; PPR, paired-pulse ratio.

**FIGURE 4:**
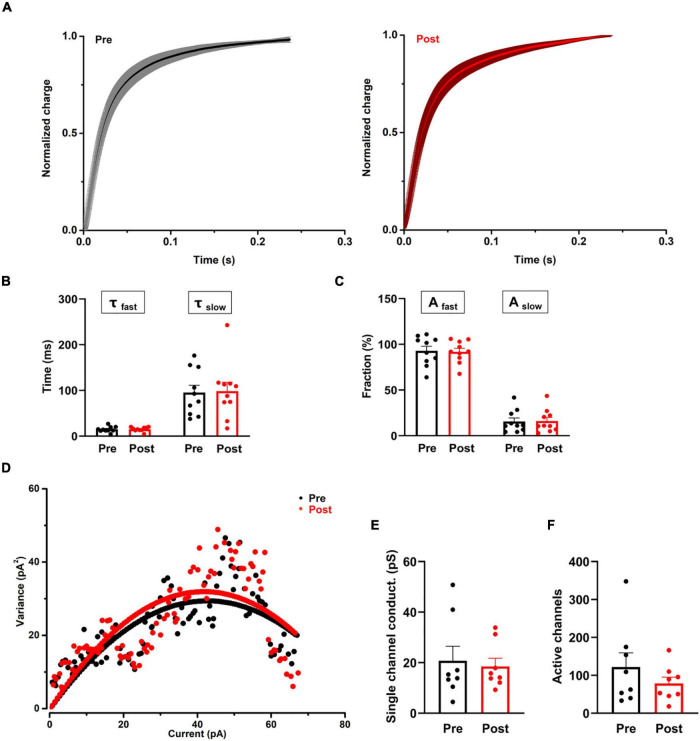
The effect of proanthocyanidins on the kinetic properties of the synaptic transmission of PBN-projecting neurons in the spinal dorsal horn. **(A–C)** Application of proanthocyanidins did not change the fast (τ_*fast*_) and slow (τ_*slow*_) decay time constants and the fast (A_*fast*_) and slow (A_*slow*_) fraction constituent in the TMR-positive spinal cord neurons. **(D–F)** The non-stationary fluctuation analysis (NSFA) indicated that bath application of proanthocyanidins affected neither the single channel conductance nor the AMPAR channel number in the TMR-positive spinal cord neurons.

**FIGURE 5:**
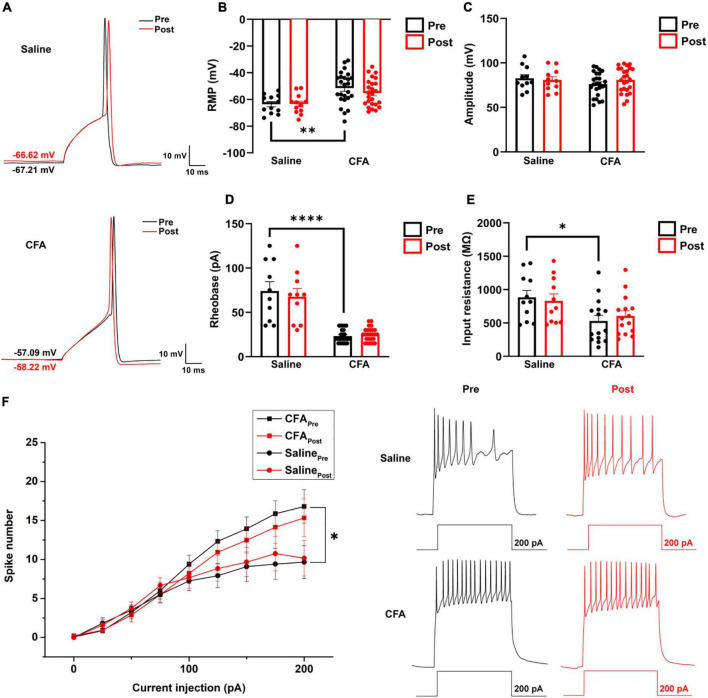
The effect of proanthocyanidins on the intrinsic properties of PBN-projecting neurons in the spinal dorsal horn. **(A)** Representative samples showing a single action potential of one PBN-projecting neuron before and after the application of proanthocyanidins in saline and CFA group, respectively. Application of proanthocyanidins did not change the resting membrane potential (RMP) **(B)**, amplitude **(C)**, rheobase **(D)**, and membrane input resistance **(E)** in the CFA or saline group. **(F)** Application of proanthocyanidins did not change the neuronal spike number in the CFA or saline group. *P < 0.05; ^**^P < 0.01; ^****^P < 0.0001. RMP, resting membrane potential; CFA, Complete Freund’s Adjuvant.

All data are shown as the mean ± SEM. Data were analyzed using paired and unpaired *t*-tests or two-way ANOVA. Unpaired *t*-tests were used for behavior test in Figure 1 and Western blotting in Figure 5. Paired *t*-tests and two-way ANOVA were used for whole-cell patch in Figures 2–4. A more detailed description has been carried out in the results section together with the statistical results. For all analyses, the criterion of significance was set at *P* < 0.05. All statistical analyses were performed using GraphPad Prism 8.4.2 software. Figures were made using GraphPad Prism 8.4.2 and OriginPro 9.1.

## Results

### Intrathecal Application of Proanthocyanidins Induces Analgesic Effects in Mice With Complete Freund’s Adjuvant Injection

First, we explored whether intrathecal (*i.t.*) injection of proanthocyanidins should change the mechanical and heat pain responses by testing the PWMT and PWTL in mice administered with CFA or saline injection. We found that the PWMT and PWTL of mice with left paw CFA (10 μl) injection were significantly lower than those of mice with the same amount of saline injection, indicating that mechanical allodynia and thermal hyperalgesia occurred in mice with CFA injection (*P* < 0.0001, *n* = 6 and 10 in the saline and CFA groups, respectively, unpaired *t*-test) (Figures 1A,B). However, *i.t.* injection of proanthocyanidins on the third (*P* = 0.003, proanthocyanidins vs. ACSF, *n* = 10 in each group, unpaired *t*-test) and seventh (*P* = 0.011, proanthocyanidins vs. ACSF, *n* = 8 in each group, unpaired *t*-test) days post-surgery significantly increased the PWMT in mice with CFA injection but not in mice with saline injection (third day: *P* = 0.56, seventh day: *P* = 0.56, proanthocyanidins vs. ACSF, *n* = 6 in each group, unpaired *t*-test) (Figure 1A). In addition, *i.t.* injection of proanthocyanidins on the third (*P* = 0.009, proanthocyanidins vs. ACSF, *n* = 8 in each group, unpaired *t*-test) and seventh (*P* = 0.002, proanthocyanidins vs. ACSF, *n* = 8 in each group, unpaired *t*-test) days post-surgery significantly increased the PWTL in mice with CFA injection but not in mice with saline injection (third day: *P* = 0.31, *n* = 8; seventh day: *P* = 0.53, *n* = 6, proanthocyanidins vs. ACSF, unpaired *t*-test) (Figure 1B). These results indicate that proanthocyanidins at the spinal cord level inhibit both mechanical and thermal pain in a mouse model of inflammatory pain induced by CFA injection.

We then tested the locomotion activities of mice using the open field test and rotary rod test. CFA injection decreased the total moving distance in the open field test (*P* = 0.004, CFA vs. saline, *n* = 6 in each group, unpaired *t*-test), which was rescued by *i.t.* injection of proanthocyanidins (*P* = 0.03, proanthocyanidins vs. ACSF, *n* = 6 in each group, unpaired *t*-test) in mice with CFA injection but not in mice with saline injection (*P* = 0.57, proanthocyanidins vs. ACSF, *n* = 6 in each group, unpaired *t*-test) (Figure 1C). Motor coordination and antifatigue ability were decreased in the rotary rod test in mice with CFA compared to those in mice with saline injection (*P* = 0.03, *n* = 8 and 10 in the saline and CFA groups, respectively, unpaired *t*-test), which were not affected by *i.t.* injection of proanthocyanidins (saline: *P* = 0.85, CFA: *P* = 0.71; proanthocyanidins vs. ACSF, *n* = 8 and 10 in the saline and CFA groups, respectively, unpaired *t*-test) (Figure 1D). These results suggest that CFA injection decreases mice’s desire to move and motor coordination ability. Application of proanthocyanidins rescues the impaired locomotion desire but not motor coordination ability.

### Bath Application of Procyanidins Decreases Excitatory Inputs to Parabrachial Nucleus-Projecting Neurons in the Spinal Cord

We next tested whether procyanidins affected the synaptic transmission and intrinsic properties of spinal noxious neurons. Since most of the projecting neurons in the spinal cord send ascending nociceptive information to the contralateral side of the lateral part of the PBN (Todd, 2010), we injected retrograde tracer TMR into the right lateral PBN to label the projecting neurons in the left spinal cord (the ipsilateral side of CFA injection) (Figure 2A). TMR retrogradely labeled (TMR^+^) neurons were primarily located in lamina I of the left spinal cord, consistent with previous reports (Todd, 2010). We then applied whole-cell patch-clamp recording of the TMR^+^ neurons and tested the sEPSC, which represents the probability of presynaptic excitatory neurotransmitter release and postsynaptic responses (Chen et al., 2015). We found that both the frequency (*P* < 0.0001) and amplitude (*P* = 0.0119) of sEPSCs were significantly increased in mice with CFA injection compared to those in mice with saline injection (*n* = 20 cells and 22 cells from 5 mice in the saline and CFA groups, respectively, unpaired *t*-test), indicating enhanced excitatory synaptic transmission to PBN-projecting spinal neurons in mice with inflammatory pain (Figures 2B,C,F,G). Bath application of proanthocyanidins (50 μM) significantly reduced the frequency (*P* = 0.005) but not the amplitude of the sEPSCs (*P* = 0.13) in the CFA group (*n* = 22 cells from 5 mice, paired *t*-test), nor did the frequency (*P* = 0.49) or amplitude (*P* = 0.97) of the sEPSCs in mice with saline injection (*n* = 20 cells from 4 mice, paired *t*-test) (Figures 2B–G). These results indicate that proanthocyanidins inhibit presynaptic glutamate inputs to PBN-projecting spinal neurons without changing AMPA receptor-mediated postsynaptic responses.

We then recorded the paired-pulse ratio (PPR) of evoked EPSCs (eEPSCs) of PBN-projecting spinal neurons to confirm the presynaptic effect of proanthocyanidins. Paired monosynaptic eEPSCs are induced by electrical stimulation of the dorsal root entry zone (Li et al., 1999) at 50 ms intervals. We found that the PPR was decreased in mice injected with CFA compared to that in mice injected with saline (*P* = 0.0091, *n* = 10 cells from 5 mice in each group, unpaired *t*-test), indicating increased peripheral inputs to PBN-projecting spinal neurons (Figures 2H,I,L). Bath application of procyanidins increased the PPR in mice with CFA injection (*P* = 0.0032, *n* = 10 cells from 5 mice, paired *t*-test) but not in mice with saline injection (*P* = 0.65, *n* = 10 cells from 5 mice, paired *t*-test) (Figures 2H–L), further confirming the presynaptic inhibitory effect of procyanidins in mice with CFA injection.

### Bath Application of Procyanidins Does Not Change the Inhibitory Inputs to Parabrachial Nucleus-Projecting Neurons in the Spinal Cord

We further recorded the sIPSC of TMR^+^ spinal neurons to determine whether procyanidins affect inhibitory inputs to PBN-projecting spinal neurons. In mice with CFA injection, the frequency (*P* = 0.003) but not the amplitude (*P* = 0.45) of the sIPSCs was decreased in comparison with those in mice with saline injection (*n* = 23 cells from 5 mice in each group, unpaired *t*-test) (Figures 3A,B,E,F), suggesting decreased inhibitory inputs to PBN-projecting spinal neurons in mice with CFA injection. We found that bath application of proanthocyanidins affected neither the frequency nor the amplitude of sIPSCs in the CFA (frequency: *P* = 0.50; amplitude: *P* = 0.82; *n* = 23 cells from 5 mice, paired *t*-test) or saline (frequency: *P* = 0.51; amplitude: *P* = 0.25; *n* = 23 cells from 5 mice, paired *t*-test) group (Figures 3A–F), suggesting that proanthocyanidins do not alter inhibitory synaptic transmission into PBN-projecting spinal neurons. These effects were confirmed by testing the PPR of the evoked IPSCs: the PPR was increased in mice with CFA injection compared to that in mice with saline injection (*P* = 0.0098, *n* = 11 and 10 cells from 5 mice in the saline and CFA groups, respectively, unpaired *t*-test); bath application of procyanidins did not affect the PPR in the CFA (*P* = 0.75, *n* = 10 cells from 5 mice, paired *t*-test) or saline group (*P* = 0.22, *n* = 11 cells from 5 mice, paired *t*-test) (Figures 3G–K).

### Bath Application of Procyanidins Does Not Affect the Kinetic Properties of the Synaptic Transmission of Parabrachial Nucleus-Projecting Neurons in the Spinal Cord

We then recorded the single eEPSCs of PBN-projecting spinal neurons to investigate whether procyanidins affect glutamate release kinetics and the properties of post-synaptic AMPA channels. We found that bath application of proanthocyanidins affected neither the decay time constants [the fast decay (τ_*fast*_): *P* = 0.87; the slow decay (τ_*slow*_): *P* = 0.81, *n* = 10 cells from 5 mice, paired *t*-test] nor the fraction constituent [the fast constituent (A_*fast*_): *P* = 0.82; the slow constituent (A_*slow*_): *P* = 0.88, *n* = 10 cells from 5 mice, paired *t*-test] in the TMR^+^ spinal neurons (Figures 4A–C), suggesting that procyanidins do not affect the pre-synaptic glutamatergic vesicle release velocity and their presynaptic inhibitory effect may be mediated by the reduced number of released vesicles. We then applied the non-stationary fluctuation analysis (NSFA) of eEPSCs to compare the unitary conductance and number of active channels in TMR^+^ spinal neurons before and after the application of procyanidins. We found that bath application of proanthocyanidins affected neither the unitary conductance (*P* = 0.79, *n* = 8 cells from 5 mice, paired *t*-test) nor the number of active channels (*P* = 0.27, *n* = 8 cells from 5 mice, paired *t*-test) (Figures 4D–F), which indicate that proanthocyanidins do not affect the number and ion conductance of post-synaptic AMPA receptors and further confirm that proanthocyanidins do not affect the amplitude of AMPA receptor-mediated EPSCs (Figure 2).

### Bath Application of Procyanidins Does Not Affect the Intrinsic Properties of Parabrachial Nucleus-Projecting Neurons in the Spinal Cord

To investigate whether procyanidins alter the intrinsic properties of PBN-projecting spinal neurons, we studied the single action potential properties and firing patterns of TMR^+^ spinal neurons before and after bath application of procyanidins. The parameters of a single action potential, such as the resting membrane potential (RMP) (*P* = 0.0026, *n* = 12 cells and 25 cells from 5 mice in the saline and CFA groups, respectively, unpaired *t*-test), rheobase (*P* < 0.0001, *n* = 10 cells and 25 cells from 5 mice in the saline and CFA groups, respectively, unpaired *t*-test) or membrane input resistance (*P* = 0.0128, *n* = 11 cells and 15 cells from 5 mice in the saline and CFA groups, respectively, unpaired *t*-test), but not the action potential amplitude (*P* = 0.18, *n* = 11 cells and 25 cells from 5 mice in the saline and CFA groups, respectively, unpaired *t*-test) was significantly different between mice with CFA or saline injection (Figures 5A–E). Application of procyanidins did not change the RMP, action potential amplitude, rheobase, and membrane input resistance in the CFA (RMP: *P* = 0.17; amplitude: *P* = 0.20; rheobase: *P* = 0.41; input resistance: *P* = 0.47, *n* = 15 or 25 cells from 5 mice, paired *t*-test) or saline group (RMP: *P* = 0.86; amplitude: *P* = 0.45; rheobase: *P* = 0.23; input resistance: *P* = 0.56, *n* = 10–12 cells from 5 mice, paired *t*-test) (Figures 5A–E). Meanwhile, the spike number induced by step depolarized current injection was increased in mice with CFA injection. However, bath application of procyanidins did not change the spike number (two-way ANOVA, *n* = 15 cells and 11 cells from 5 mice in the CFA and saline groups, respectively) (Figure 5F).

### Proanthocyanidins Inhibit Phosphorylated Activation of the PI3K/Akt/mTOR Signaling Pathway in the Dorsal Root Ganglia Neurons

**FIGURE 6:**
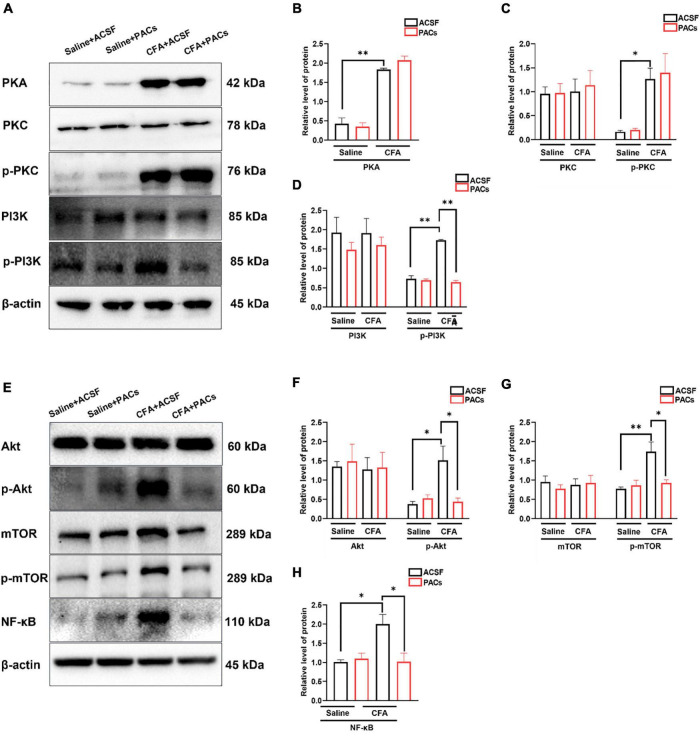
The role of proanthocyanidins in the PI3K/Akt/mTOR signaling pathway. **(A)** Representative bands of PKA, PKC/p-PKC, and PI3K/p-PI3K. **(B–D)** Application of proanthocyanidins reversed the activation of p-PI3K but not PKA, PKC/p-PKC, or PI3K in the CFA group. **(E)** Representative bands of Akt/p-Akt, mTOR/p-mTOR, and NF-κB. **(F–H)** Application of proanthocyanidins reversed the activation of p-Akt, p-mTOR, and NF-κB but not Akt and mTOR in the CFA group. *P < 0.05; ^**^P < 0.01. CFA, Complete Freund’s Adjuvant; ACSF, artificial cerebrospinal fluid; PACs, proanthocyanidins.

Since proanthocyanidins inhibit excitatory peripheral inputs to PBN-projecting fibers, we explored whether proanthocyanidins affected the expression of intracellular signaling molecules in the DRG neurons. We first assessed the expression of PKA, PKC, and PI3K proteins in the DRG neurons after 30 min of incubation of the DRG with procyanidins-containing ACSF. We found that the protein expression of PKA (*P* = 0.009), phosphorylated PKC (p-PKC) (*P* = 0.039), and phosphorylated PI3K (p-PI3K) (*P* = 0.0086) was significantly increased in mice with CFA injection compared to those with saline injection (*n* = 8 mice in each group, unpaired *t*-test) (Figures 6A–D). In the case of incubation with proanthocyanidins, the increased p-PI3K expression (*P* = 0.003), but not the PKA (*P* = 0.44) and p-PKC (*P* = 0.97) expression, was reversed (proanthocyanidins vs. ACSF, *n* = 8 mice in each group, unpaired *t*-test). This suggests that proanthocyanidins primarily inhibit phosphorylated activation of the PI3K pathway in the DRG neurons in mice with CFA injection.

We then examined whether proanthocyanidins affected the expression of downstream molecules in the PI3K pathway. Compared to the saline group, expression of p-Akt (*P* = 0.014), p-mTOR (*P* = 0.007), and NF-κB (*P* = 0.023) proteins was increased in CFA-injected mice (*n* = 8 mice in each group, unpaired *t*-test) (Figures 6E–H). In the case of incubation with proanthocyanidins, the increased expression of p-Akt (*P* = 0.019), p-mTOR (*P* = 0.02), and NF-κB (*P* = 0.025) was reversed (*n* = 8 mice in each group, paired *t*-test).

## Discussion

Proanthocyanidins are a type of pigment in plants. They are common in the flowers, nuts, fruits, bark, and seeds of various plants. Berries and fruits are the best sources of proanthocyanidins for human intake. In the stomach environment, 3–6 U of proanthocyanidins will be hydrolyzed into free catechins and catechin dimers and then absorbed into the blood (Spencer et al., 2000). These results indicate the possible application of procyanidins as raw materials in medicine, health care products, food, cosmetics, and other fields.

In the area of human health, proanthocyanidins exert diverse protective effects on the human body. Oligomeric proanthocyanidin complexes have antioxidant, antibacterial, antiviral, anticancer, anti-inflammatory, antiallergic, and vasodilator effects (Fine, 2000; Bao et al., 2015). The protective effects have also been observed in neurodegenerative diseases. Previous research indicated that the neuroprotective effect of proanthocyanidins on Alzheimer’s disease (AD) is mediated by inhibiting amyloid β aggregation, reducing amyloid β production, and preventing small amyloid β neurotoxicity in the mouse brain (Li et al., 2016). Proanthocyanidins’ analgesic effect has also been reported. Gavage of plant extracts rich in proanthocyanidins significantly reduces acute inflammatory pain in a variety of mouse inflammatory pain models induced by carrageenan, capsaicin, cinnamaldehyde, or formalin (Fongang et al., 2017). Gavage of proanthocyanidins significantly alleviates the hyperalgesia caused by abnormal peripheral nerve function in type 2 diabetic rats (Ding et al., 2014) and sciatic nerve injury mice (Pan et al., 2018). In our present study, we also found that intrathecal application of proanthocyanidins (20 μg) induced a significant analgesic effect in mice with inflammatory pain. Our present results and previous works all suggest that proanthocyanidins are good candidates for clinical analgesic agent exploitation, especially considering that proanthocyanidins are easily absorbed by oral intake.

Studies of the potential mechanism of proanthocyanidins in the central nervous system and in spinal analgesia are very limited, and the involvement of ion channels in proanthocyanidins’ effect may be considered. In our experiments, proanthocyanidins did not affect the decay kinetics of the eEPSC and the post-synaptic AMPA receptor ion conduction. We thus propose that proanthocyanidins should not affect the properties of pre- and post-synaptic ion channels in the spinal cord. However, it’s shown that proanthocyanidins induce hyperpolarization of rat aorta endothelial cells *via* multiple activations of K^+^ channels and play a protective role in Ca^2+^ influx into endothelial cells (Byun et al., 2012). Proanthocyanidins are also reported to help maintain the intracellular Ca^2+^-homeostasis after NMDA receptor overactivation, protecting neurons from Ca^2+^-induced adverse effects (Franchi et al., 2020). Besides that, restricting Ca^2+^ influx can reduce the activation of PI3K and Akt, and then produce a protective effect on glutamate-induced excitotoxicity. We thus think that the effect of proanthocyanidins in the properties of ion channels, especially Ca^2+^ channels, should be further investigated in the future.

In addition, studies reporting that the regulation of synaptic plasticity by proanthocyanidins cannot be ignored. Procyanidins promote basic synaptic transmission and long-term synaptic potentiation in hippocampal slices, and significantly improve the cognitive impairment caused by AD (Wang et al., 2012), through inhibiting oxidative stress and retaining AKT and ERK activity (Gao et al., 2020). In our results, procyanidins also inhibited the short-term plasticity of the synaptic transmission in the spinal cord, which may be mediated by affecting the pre-synaptic vesicle release, since procyanidins did not affect the number and ion conductance of post-synaptic AMPAR. However, whether and how procyanidins affect the long-term plasticity of the spinal cord is not known yet and is interesting for future investigation.

In previous works, it has been shown that the mechanisms of proanthocyanidin analgesia in peripheral nerves may primarily derive from strong antioxidants (Mao et al., 2015), anti-swelling (Cordeiro et al., 2016), and inhibition of Ca^2+^-ATPase activities (Ding et al., 2014). Although one study reports that the gavage of proanthocyanidins inhibits the activity of spinal MMP-9 and MMP-2 (Pan et al., 2018), whether proanthocyanidins affect the synaptic transmission of nociceptive information in the central nervous system, which is the primary pathological change in central hypersensitization, remains obscure. Our study is the first to observe the presynaptic inhibitory effect of proanthocyanidins on spinal cord neurons projecting to supraspinal areas in mice with inflammatory pain, providing preliminary evidence for the central application of proanthocyanidins in the field of analgesia.

We also noticed that proanthocyanidins are beneficial to exercise management. This is in consistent with previous works that intraperitoneal injection of a moderate dose of proanthocyanidins increases the exercise capacity of rats (Moreira et al., 2010). We found that proanthocyanidins did not rescue the damaged exercise capacity in the rotary rod experiment but rescued the reduced movement distance in the open field test, in mice with CFA injection. Our point of view for these results is that proanthocyanidins have an analgesic effect but do not impact the animal’s ability to move, instead of increasing the animal’s desire for movement. Under the unique environment of the rotary rod, plantar swelling-caused damage of exercise ability should not be reduced by *i.t.* injection of proanthocyanidins. On the contrary, in the open field experiment, application of proanthocyanidins reduces the pain and negative emotion and increases animals’ motivation for exploration.

In summary, we find that proanthocyanidins, as food-grade substances, have a potent relieving effect on inflammatory pain in animals with CFA injection, through inhibition of the nociceptive inputs to the PBN-projecting spinal neurons. However, although their application in the treatment of inflammatory pain is of potential interests, many questions need to be clarified, such as their possible effect on the long-term synaptic plasticity and ion channels, as well as the underlying molecular mechanisms in addition to inhibition of PI3K pathways and their effects on different groups of DRG neurons sending projections to spinal neurons.

## Data Availability Statement

The raw data supporting the conclusions of this article will be made available by the authors, without undue reservation.

## Ethics Statement

The animal study was reviewed and approved by the Animal Care and Use Committee for Research and Education of the Fourth Military Medical University.

## Author Contributions

ZW, YL, and TC: concept and design. HF, ZW, DZ, JG, MX, MZ, and HD: data collection analysis and interpretation. HF, ZW, and TC: manuscript writing. HF, ZW, DZ, JG, MX, MZ, HD, and TC: final approval of the manuscript. All authors contributed to the article and approved the submitted version.

## Conflict of Interest

The authors declare that the research was conducted in the absence of any commercial or financial relationships that could be construed as a potential conflict of interest.

## Publisher’s Note

All claims expressed in this article are solely those of the authors and do not necessarily represent those of their affiliated organizations, or those of the publisher, the editors and the reviewers. Any product that may be evaluated in this article, or claim that may be made by its manufacturer, is not guaranteed or endorsed by the publisher.
